# Mycoremediation of Benzo[*a*]pyrene by *Pleurotus ostreatus* in the presence of heavy metals and mediators

**DOI:** 10.1007/s13205-013-0148-y

**Published:** 2013-06-07

**Authors:** Sourav Bhattacharya, Arijit Das, Kuruvalli Prashanthi, Muthusamy Palaniswamy, Jayaraman Angayarkanni

**Affiliations:** 1 Department of Microbiology, Karpagam University, Coimbatore, 641021 Tamil Nadu India; 2 Department of Microbiology, Genohelix Biolabs, Centre for Advanced Studies in Biosciences, Jain University, Bangalore, 560019 Karnataka India; 3 Department of Biotechnology, Genohelix Biolabs, Centre for Advanced Studies in Biosciences, Jain University, Bangalore, 560019 Karnataka India; 4 Department of Microbial Biotechnology, Bharathiar University, Coimbatore, 641046 Tamil Nadu India

**Keywords:** Benzo[*a*]pyrene, *Pleurotus ostreatus*, Degradation, Heavy metals, Mediators

## Abstract

Benzo[*a*]pyrene is considered as a priority pollutant because of its carcinogenic, teratogenic and mutagenic effects. The highly recalcitrant nature of Benzo[*a*]pyrene poses a major problem for its degradation. White-rot fungi such as *Pleurotus ostreatus* can degrade Benzo[*a*]pyrene by enzymes like laccase and manganese peroxidase. The present investigation was carried out to determine the extent of Benzo[*a*]pyrene degradation by the PO-3, a native isolate of *P*. *ostreatus,* in the presence of heavy metals and ligninolytic enzyme mediators. Modified mineral salt medium was supplemented with 5 mM concentration of different heavy metal salts and ethylenediaminetetraacetic acid. Vanillin and 2,2′-azinobis-(3-ethylbenzothiazoline-6-sulfonate) (1 and 5 mM) were used to study the effect of mediators. Results indicated that *P*. *ostreatus* PO-3 degraded 71.2 % of Benzo[*a*]pyrene in the presence of copper ions. Moderate degradation was observed in the presence of zinc and manganese. Both biomass formation and degradation were severely affected in the presence of all other heavy metal salts used in the study. Copper at 15 mM concentration supported the best degradation (74.2 %), beyond which the degradation progressively reduced. Among the mediators, 1 mM 2,2′-azinobis-(3-ethylbenzothiazoline-6-sulfonate) supported 78.7 % degradation and 83.6 % degradation was observed under the influence of 5 mM vanillin. Thus, metal ion like copper is essential for better biodegradation of Benzo[*a*]pyrene. Compared to synthetic laccase mediator like 2,2′-azinobis-(3-ethylbenzothiazoline-6-sulfonate), natural mediator such as vanillin may play a significant role in the degradation of aromatic compounds by white-rot fungi.

## Introduction

Benzo[*a*]pyrene (B*a*P), a representative of high molecular weight polycyclic aromatic hydrocarbon (HMW PAH), consists of five fused benzene rings and is of environmental concern since it behaves as a potent teratogen, mutagen and carcinogen. Its high molecular weight and extremely low water solubility reduce its bioavailability, thus making it resistant to microbial degradation (Rentz et al. 2008). Together with other PAHs, B*a*P is commonly formed under natural conditions like volcanic eruptions and forest fires and by anthropogenic activities like the pyrolysis and incomplete combustion of fossil fuels (Li et al. 2009).

Different strategies for removal of B*a*P and other highly recalcitrant compounds from contaminated sites include chemical oxidation, photolysis and biodegradation. Among all these clean up strategies, involvement of microorganisms is a subject of intense research and gains a cutting edge advantage for being less expensive and environment friendly (Chatterjee et al. 2008).

In nature there exists a great diversity of microorganisms capable of PAH degradation. Bacteria are capable of degrading the low molecular weight PAH, such as naphthalene, acenaphthene and phenanthrene and relatively few genera have been observed to degrade the HMW PAHs, such as B*a*P (Juhasz and Naidu 2000). However, several white-rot fungi (*Phanerochaetechrysosporium*, *Coriolusversicolor*, *Strophariacoronilla*, *Pleurotusostreatus*, *Irpex lacteus* and *Bjerkandera adusta*) that synthesizes extracellular lignin modifying enzymes like lignin peroxidases (LiP), manganese peroxidases (MnP), laccases and other oxidases can oxidize B*a*P and similar HMW PAHs with up to six aromatic rings (Pointing 2001).

Though many studies on microbial degradation of B*a*P have been performed, literature on the effect of simultaneous existence of different pollutants groups on the extent of B*a*P bioremediation is scanty. PAH contaminated sites near industrial land are often accompanied by the presence of high levels of heavy metals (Sun et al. 2010). Heavy metals may be toxic for white-rot fungi and may have a negative effect on the activity of their ligninolytic enzymes (Bamforth and Singleton 2005). Occurrence of both organic and inorganic contaminants on the same site can therefore challenge the effectiveness of bioremediation technologies (Roy et al. 2005).

Among the oxidases produced by white-rot fungi, laccases have relatively low redox potential (0.4–0.8 V), belong to a group of polyphenol oxidases containing copper atoms in the catalytic site and usually called multicopper oxidases (Palmer et al. 1999). Laccases are responsible for lignin degradation in nature. This enzyme lacks substrate specificity and is thus capable of degrading a wide range of non ligninolytic compounds including certain xenobiotics like industrial colored waste water, oil and oil products, chlorinated compounds and PAHs (Abo-State et al. 2011).

Laccase limitations, owing to their relative low redox potential, have been overcome using redox mediators like 2,2′-azinobis-(3-ethylbenzothiazoline-6-sulfonate) (ABTS) or 1-hydroxybenzotriazole (HBT) which improves the oxidation of PAHs by laccase (Johannes and Majcherczyk 2000). However, the use of these synthetic mediators makes the process expensive and may result in the development of toxic end products. In contrast, some products generated from lignin degradation could act as natural redox mediators of laccase. The use of natural compounds provides a low cost, ecofriendly and toxicity less process (Moldes et al. 2008).

Therefore the objective of the study is to evaluate the effect of heavy metals on the extent of B*a*P degradation and the potential of synthetic and natural mediators to promote B*a*P transformation by the native isolate, *P. ostreatus* PO-3.

## Materials and methods

### Chemicals and reagents

HPLC grade B*a*P standard (98 % pure) was procured from Spectrochem Pvt. Ltd., Mumbai, India. Other fine chemicals used were procured from SRL Chemicals, India and were of the highest purity and analytical grade.

### Effect of heavy metals and mediators

To study the effect of heavy metals and mediators on the degradation of B*a*P (10 μg/ml of the mineral salt medium), the PO-3 isolate of *P. ostreatus* from our previous study was used (Bhattacharya et al. 2012a). The optimized nutrients and surfactant from our earlier study were added to the modified mineral salt medium (devoid of CuSO_4_ and MnSO_4_) with the following composition (g/L): (NH_4_)_2_HPO_4_, 05; KH_2_PO_4_, 0.8; K_2_HPO_4_, 0.3; MgSO_4_·7H_2_O, 0.3; CaCl_2_·2H_2_O, 0.055; Thiamine, 1 ml (2 mg/ml). The optimized physical parameters were also maintained (Bhattacharya et al. 2012b).

The effect of heavy metals and ethylenediaminetetraacetic acid (EDTA) on B*a*P degradation and biomass formation was studied by incorporating salts of different heavy metals (CuSO_4_, ZnSO_4_, MnSO_4_, AgNO_3_, CdCl_2_, and HgCl_2_) at 5 mM concentration to the modified mineral salt medium. The effect of different concentrations (5, 10, 15, 20, 25, 30 and 50 mM) of selected heavy metal salt that resulted in highest B*a*P degradation and fungal growth was analyzed. ABTS and vanillin (1 and 5 mM) were used as mediators in the study. Broth without the heavy metals or the mediators served as the control.

### Analytical methods

#### Extraction of residual B*a*P

Following incubation, the extraction of residual B*a*P was performed using a modified method of Capotorti et al. (2004). The concentrated extract was subjected to high performance liquid chromatography analysis.

#### High performance liquid chromatography analysis

The condensed sample was subjected to filtration using 0.25 μ nitrocellulose membrane filter. The working standard solution of B*a*P (concentration of 5 μg/ml) was prepared using 80:20 (v/v) of acetonitrile: water. 20 μl of the eluate containing 0.1 μg of the standard B*a*P was injected into the HPLC system (Waters, USA, model number-2487, with dual λ absorbance UV detector and binary pump system, model number-1525). A reverse phase, C-18 column (150 × 4.6 mm) was used. The mobile phase used was acetonitrile: water (80:20 v/v). The flow rate was maintained at 1 ml/min. The concentration of the B*a*P standard solution was determined at 254 nm. Area under the absorbance peak was used to estimate the percentage of degradation using a formula:

[(*C*_i_ − *C*_f_)/*C*_i_]*100, where *C*_i_ is the initial concentration of B*a*P and *C*_f_ is the final concentration of B*a*P.

### Statistical analysis

The effect of each parameter was studied in triplicate and the data are graphically presented as the mean ± S.D. of triplicates (*n* = 3). All the graphs have been prepared using Microsoft Excel 2007.

## Results and discussion

Mycoremediation refers to fungal degradation or transformation of hazardous organic contaminants to less toxic compounds (Sasek et al. 2003). *P. ostreatus* is a white-rot basidiomycete fungus whose ligninolytic enzyme machinery consists of laccase and manganese peroxidase. It is often considered a model organism for the degradation of xenobiotics because of its ability to colonize soil and remains unaffected by the presence of indigenous microflora, as well as its excellent performance in biodegradation studies. Several isoenzymes of laccase from *P. ostreatus* have been reported and many have been purified for their potential applications. A range of compounds, including aromatic substrates of laccase that regulate the enzyme activity is well documented (Palmieri et al. 1997).

### Effect of heavy metals on B*a*P degradation

Heavy metals often act as important modulators for enzyme activity. Many of these heavy metals are present in the environment either naturally (Cu^2+^) or may gain their entry as a result of anthropogenic activities (Cd^2+^, Hg^2+^, Pb^2+^). Polluted sites often contain high concentrations of heavy metals. Co-contamination with heavy metals may be a road block for in situ biodegradation of xenobiotics like PAHs or polychlorinated biphenyls (Koeleman et al. 1999).

**Fig. 1:**
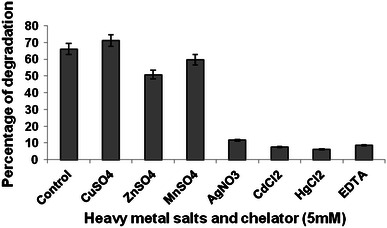
Effect of heavy metal salts and chelator on degradation of B*a*P by *P. ostreatus*

**Fig. 2:**
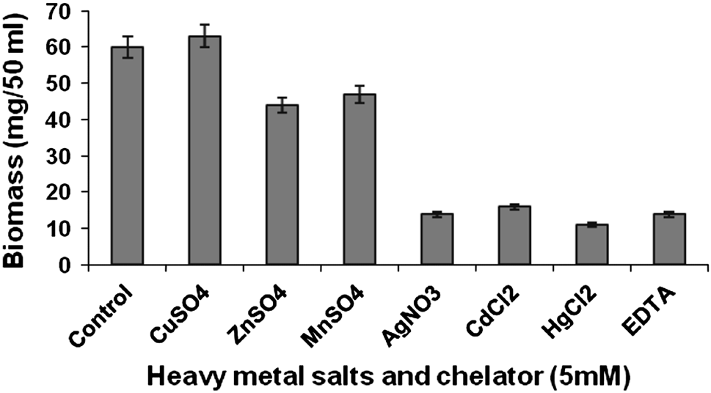
Effect of heavy metal salts and chelator on *P. ostreatus* biomass formation

Maximum degradation and biomass formation by *P. ostreatus* PO-3 resulted in the presence of Cu^2+^ ions (71.2 % and 63 mg of biomass/50 ml of broth). Moderate degradation resulted from the presence of Zn^2+^ and Mn^2+^. Both biomass formation and degradation were severely affected in the presence of Ag^2+^, Cd^2+^, Hg^2+^ and EDTA (Figs. 1, 2).

The present result demonstrating the increase in the extent of B*a*P degradation by *P. ostreatus* PO-3 following the addition of Cu^2+^ may be attributed to the increase in the laccase activity, which may in turn be due to both increased laccase production and the stabilization of the enzyme in an extracellular environment. Copper acting as a strong laccase inducer in *P. ostreatus* was reported earlier (Palmieri et al. 2000).

While characterizing the laccase from *Myrothecium verrucaria* NF-05, the resultant large extent activation of laccase activity in the presence of Cu^2+^ might be caused by the filling of type-2 copper binding sites with Cu^2+^ (Sulistyaningdyah et al. 2004).

Cadmium is a non-essential heavy metal. The presence of cadmium severely affected *P. ostreatus* PO-3 biomass formation probably due to the induction of oxidative stress. B*a*P degradation was also hindered by the presence of cadmium, since the sensitivity of fungi toward Cu^2+^ and Cd^2+^ changes with time. Laccase induction only occurs when cadmium is added during the later stages of growth. Parallel to our result, when cadmium was added earlier than 12 days, the activation of laccase was decreased. Ag^2+^, Hg^2+^ and Pb^2+^ also decreased the activity of laccase (Baldrian and Gabriel 2002). Mercury has high affinity for thiol groups in proteins, which might have lead to the inactivation of enzymes involved in the degradation (Baldrian et al. 2000).

### Effect of different concentration of Cu^2+^ on B*a*P degradation

Since copper is a biogenic metal present in the environment, its concentration may have an important role in enzyme performance and stability. Copper requirement by microorganisms is usually satisfied by very low concentrations of the metal (1–10 mM). Copper present in higher concentration proves to be extremely toxic to microbial cells (Tychanowicz et al. 2006), although reports of copper tolerance by some fungi exist (De Groot and Woodward 1999).

**Fig. 3:**
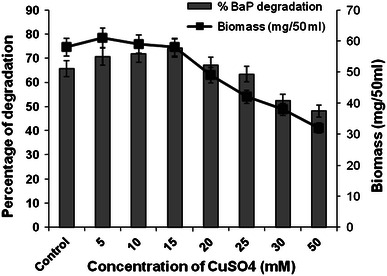
Effect of different concentrations of CuSO_4_ on B*a*P degradation and *P. ostreatus* biomass formation

Result from the present study shows that greater biomass was formed in lower concentrations as compared to higher concentrations of Cu^2+^. Appreciable biomass and B*a*P degradation were observed up to the presence of 15 mM of Cu^2+^ supporting 74.2 % B*a*P degradation. However, higher concentrations of copper progressively decreased biomass and B*a*P degradation by *P. ostreatus* PO-3 (Fig. 3).

An early research reports similar finding, where higher Cu^2+^ concentration resulted in the reduced biomass formation. The probable reason for reduction in biomass formation at elevated level of Cu^2+^ may be attributed to the induced oxidative stress similar to that of cadmium (Baldrian et al. 2000). Similarly, Tychanowicz et al. (2006) reported that in the culture where copper was present at 10.0–25.0 mM concentrations, laccase activity increased up to eightfold when compared to the control cultures.

### Effect of mediators on B*a*P degradation

It is well quoted in literature that many species of white-rot fungi, including *Pleurotus* sp, produce laccases that can efficiently degrade HMW PAHs such as B*a*P (Juhasz and Naidu 2000). The laccase-catalyzed reaction depends on monoelectronic oxidation, which transforms the substrates to corresponding reactive radicals. With the incorporation of special compounds called mediators that act as a single electron donor and activator of the enzymes, oxidation rates of the enzyme can be enhanced (Morozova et al. 2007).

Synthetic mediators like ABTS and HBT are commonly used in degradation studies involving laccases and other lignin-modifying enzymes. Several natural phenolic compounds produced in the process of lignin depolymerization like vanillin, acetovanillone, acetosyringone, syringaldehyde, 2,4,6-trimethylphenol, *p*-coumaric acid, ferulic acid, and sinapic acid may also act as alternative mediators for the laccase mediated bioremediation of HMW PAHs by white-rot fungi (Cañas et al. 2007).

**Fig. 4:**
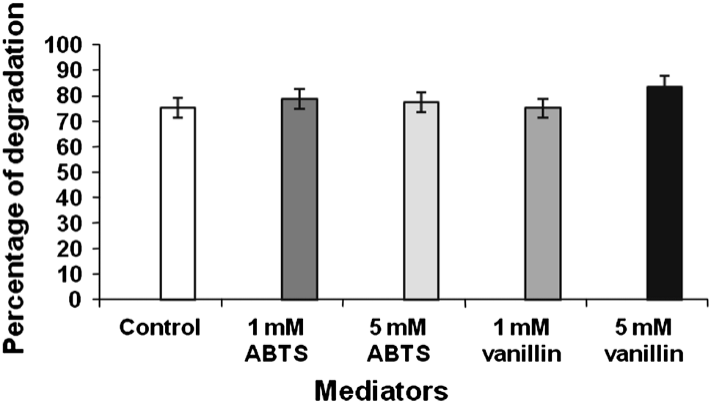
Effect of ABTS and vanillin on degradation of B*a*P by *P. ostreatus*

**Fig. 5:**
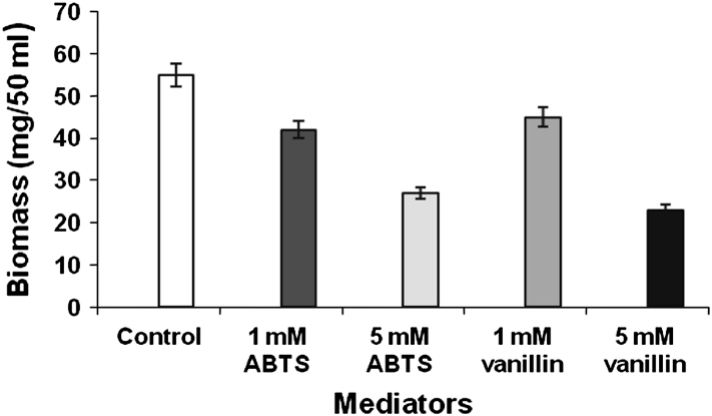
Effect of ABTS and vanillin on *P. ostreatus* biomass formation

In our study, both ABTS (artificial mediator) and vanillin (natural mediator) demonstrated positive effect on the degradation as compared to that of the control (Fig. 4). ABTS proved to be superior at 1 mM concentration (78.7 % and 42 mg of biomass/50 ml of broth). Vanillin supported better degradation (83.6 %) at 5 mM concentration. Both the mediators affected the biomass formation (Fig. 5).

Similar result was observed previously, wherein lignin-related phenolic compounds acted as laccase mediators and resulted in a significant degradation of PAH. Vanillin, acetovanillone and ferulic acid significantly promoted anthracene and B*a*P transformation by *Pycnoporus cinnabarinus* laccase (Cañas et al. 2007). The probable reason could be the structural similarities of these recalcitrant aromatic pollutants to that of lignin network.

Earlier, *Pycnoporus cinnabarinus* laccase without mediator oxidized only 12 % of B*a*P in 24 h. The presence of the natural compounds like vanillin, acetovanillone, 2,4,6-trimethylphenol and *p*-coumaric acid notably promoted transformation of B*a*P by laccase (Camarero et al. 2005). Positive effect has also been found by joining the artificial mediator ABTS and vanillin during pentachlorophenol transformation by laccase (Jeon et al. 2008).

Of the phenols tested, 4-hydroxybenzaldehyde and vanillin were most inhibitory to *Pleurotus sajor*-*caju* growth, and cultures supplemented with these two compounds also exhibited large increase in laccase-specific activity when compared to the unsupplemented controls (Lo et al. 2001).

Naturally occurring phenols such as syringaldehyde, vanillic acid, vanillin, and *p*-coumaric acid proved to be important laccase mediators effectively transforming cyprodinil up to 70 % (Kang et al. 2002). The positive effect of the phenolic compounds derived from the lignin polymer and constituents of humic acids on the oxidative transformation of widespread pollutants such as chlorinated phenols had already been demonstrated (Park et al. 1999).

**Fig. 6:**
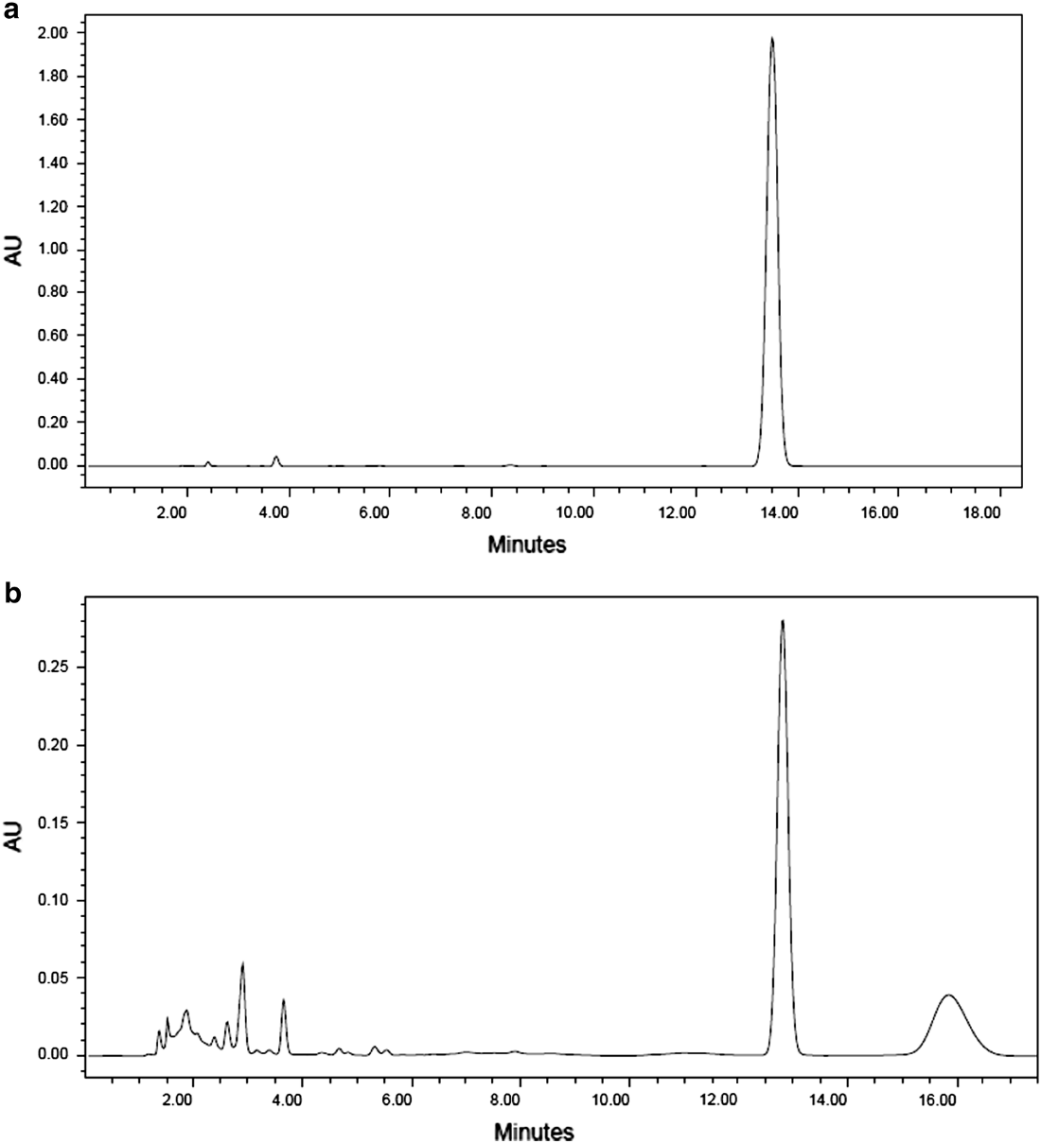
HPLC chromatogram of B*a*P **a** control, **b** after degradation

HPLC analysis reveals biodegradation of B*a*P by *P. ostreatus* PO-3 under optimized media and growth conditions (Fig. 6a, b).

## Conclusions

Various metals may be present in a contaminated site alongside the PAHs. The results showed varied levels of B*a*P degradation in the presence of heavy metals. Improved biodegradation in case of copper availability can be correlated to the activation and stabilization of the produced laccase by *P. ostreatus* PO-3.

Natural mediators may play an important role in the degradation of PAH by white-rot fungi. In future, lignin-derived mediator like vanillin may act as an alternative to synthetic mediator like ABTS. Besides improving the working potential of the ligninolytic enzymes, these natural mediators are released in large amount as a result of the microbial degradation of lignocellulose and are present in humus as common secondary plant metabolites. Vanillin and similar mediators would thus effectively reduce the cost of the bioremediation process.
